# Novel *bla*CTX-M variants and genotype-phenotype correlations among clinical isolates of extended spectrum beta lactamase-producing *Escherichia coli*

**DOI:** 10.1038/s41598-019-39730-0

**Published:** 2019-03-12

**Authors:** Ahmed A. Ramadan, Neveen A. Abdelaziz, Magdy A. Amin, Ramy K. Aziz

**Affiliations:** 1 Department of Microbiology and Immunology, Faculty of Pharmacy, Ahram Canadian University, Giza, Egypt; 2 Department of Microbiology and Immunology, Faculty of Pharmacy, Cairo University, Cairo, Egypt; 3 Center for Microbiome and Genome Research, Cairo University, Cairo, Egypt

## Abstract

The rapid emergence of multiresistant microbial pathogens, dubbed superbugs, is a serious threat to human health. Extended spectrum beta lactamase (ESBL)-producing *Escherichia coli* is a superbug causing worldwide outbreaks, necessitating timely and accurate tracking of resistant strains. Accordingly, this study was designed to investigate the spread of ESBL-producing *Escherichia coli* isolates, to analyze the effect of different genotypic and phenotypic factors on *in vitro* resistance patterns, and to assess the diagnostic value of commonly used ESBL genetic markers. For that purpose, we cultured 250 clinical isolates and screened their susceptibility to beta-lactam antibiotics. Among 12 antibiotics screened, only imipenem seems to have remained resilient. We subsequently analyzed the ESBL phenotype of *Escherichia coli* isolates and examined potential associations between their resistance phenotypes and patient-related factors. ESBL genotyping of 198 multiresistant isolates indicated that 179 contained at least one *bla*_CTX-M_ gene. As we statistically dissected the data, we found associations between overall resistance and body site / type of disease. Additionally, we confirmed the diagnostic value of testing both *bla*_CTX-M-1_ and *bla*_CTX-M-15_ in providing better prediction of overall resistance. Finally, on sequencing the amplification products of detected *bla*_CTX-M_ genes, we discovered two novel variants, which we named *bla*_CTX-M-14.2_ and *bla*_CTX-M-15.2._

## Introduction

The emergence of antibiotic resistance is an increasingly alarming public health threat, since it undermines the efficacy of antibiotic treatment^1^, and is expected to be the leading cause of global mortality by 2050, possibly exceeding cancer^2^. In *Escherichia coli (E.coli)*, one of the best-studied microbes, resistance to multiple classes of antibiotics is becoming more common, owing to the overuse and misuse of antibiotics, and pan-resistant strains (those resistant to all known antibiotics) are being increasingly reported^3^.

Beta-lactam antibiotics are among the most frequently prescribed antimicrobials worldwide. Lately, the emergence of resistance to these agents has dramatically increased. Resistance of enterobacteria to beta-lactam antibiotics is usually caused by intrinsic beta-lactamases that hydrolyze the beta-lactam ring of these antibiotic molecules. This problem was initially solved by the introduction of extended spectrum cephalosporins as therapeutic agents in the mid-1980s. Subsequently, however, extended spectrum beta-lactamases (ESBLs) emerged in numerous hospitals worldwide. These enzymes confer resistance to extended spectrum cephalosporins and related oxyimino-β-lactams (ceftazidime, cefotaxime and aztreonam), but are predominantly sensitive to carbapenems, cephamycins and beta-lactamase inhibitors, such as clavulanic acid^4^.

Resistance to beta-lactams is dramatically increasing in *E. coli* strains causing community-acquired infections, or health care-associated urinary tract and intra-abdominal infections. The proportion of such resistant strains exceeds 50% in developing countries likely because of the extensive and exaggerated clinical use of antibiotics^5^. Furthermore, a worldwide increase in resistance to extended-spectrum cephalosporins in Enterobacteriaceae, mainly *E. coli*, has led to an investigation of mechanisms involved in this resistance. Production of ESBLs was found to be the major mechanism, and among ESBLs, CTX type M variants are more common than others^6^.

ESBLs are believed to have originated from point mutations in the beta-lactamase (*bla*)-encoding genes belonging to the TEM and SHV types. As of Feb 2018, over 223 TEM types and 193 SHV types have been listed in public databases (e.g., https://www.lahey.org/Studies), albeit not all of them have ESBL phenotype^7^. Other widespread ESBLs that are neither TEM nor SHV-like are CTX-M-β-lactamases^8^. These are ESBLs belonging to Ambler’s class A / Bush’s group 2be^9^. To date, over 172 CTX-M types have been identified and described (https://www.lahey.org/studies/other.asp). They have been grouped into five clusters (CTX-M-1, CTX-M-2, CTX-M-8, CTX-M-9, and CTX-M-25), named after the first member of each group^10^. Whereas TEM and SHV enzymes are believed to have vertically evolved via point mutations in the parent enzymes, CTX-M enzymes may have evolved via horizontal transfer. Their origin is hypothesized to be the chromosomal *bla* genes of *Kluyvera* spp., from which they may have been mobilized into plasmids, perhaps via transposons. The diversity of current CTX-M family members, though, is most likely due to subsequent mutations and recombination events^9^.

In Egypt, like in many lower middle-income and low-income countries, antibiotics are often obtained from local pharmacies without prescription, and antibiotic policies are only available in a few hospitals in large cities but not in small towns or rural areas. Thus, the spread of multi-resistant microbes is a higher risk, and the danger of the emergence of pan-resistant superbugs is imminent. Individuals at high risk of these superbugs are children, elderly, and immunocompromised patients, especially those in hospitals.

Here, we launched a study to screen clinical enterobacterial isolates from patients admitted to different hospitals in Cairo, Egypt, for the spread of CTX-M ESBL-encoding genes and investigate phenotypic and genotypic factors affecting their final resistance status. Moreover, we sequenced the isolated genes from ~50% of the *bla*_CTX-M_–positive isolates to determine their *bla*_CTX-M_ genotypes. Out of 250 enterobacterial isolates, 198 beta lactam-resistant *E. coli* strains were identified, 179 of which carried variants of the *bla*_CTX-M_ genes. Having sequenced 88 of these genes, we report two novel variants of *bla*_CTX-M-14_ and *bla*_CTX-M-15_.

## Results

### Distribution of the clinical isolates

The study started by screening 250 clinical isolates recovered from different specimens (urine, stool, blood, pus, wounds, swabs, and drains) for antibiotic-resistant *Escherichia coli*. Out of the screened isolates, 198 were confirmed to be *E. coli* by standard laboratory methods.

**Figure 1:**
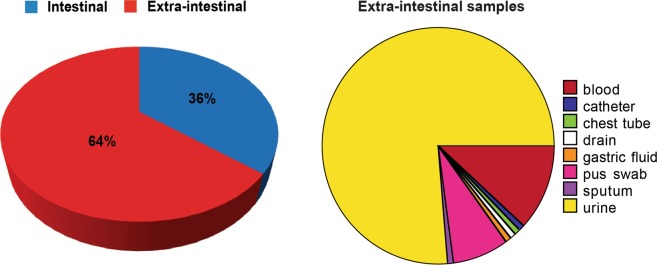
Distribution of the isolates according to their origin. Pie charts showing the relative distribution of intestinal and extra-intestinal isolates, as well as the detailed sites of isolation of the latter (Created by Data Desk version 6.3, Ithaca, NY, USA).

Among these 198 *E. coli* isolates, 71 were intestinal (36%) and 127were extra-intestinal (64%). Most extra-intestinal isolates were recovered from urine, but there were also isolates from blood, pus, and catheters (Fig. 1).

### *In vitro* antibiotic susceptibility pattern of the isolates

**Figure 2:**
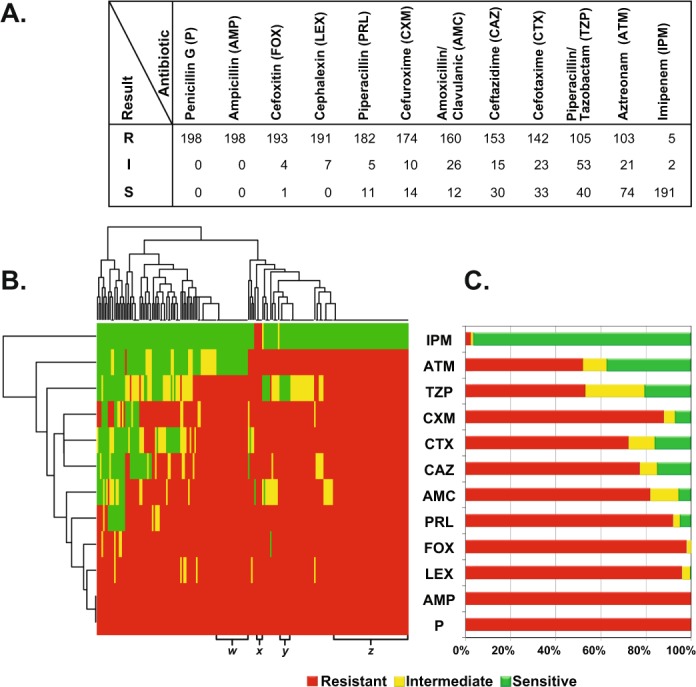
Resistance phenotypes of all 198 *E. coli* isolates to 12 beta-lactams. (**A**) Tabular representation of the results of sensitivity tests, interpreted and classified into resistant (R), intermediate (I) and sensitive (S) according to CLSI^24^. (**B**) A transposed heatmap representing the resistance phenotypes of each isolate according to the color code shown in the figure key (red = resistant; yellow = intermediate; and green = sensitive). Isolates are shown on the X-axis and antibiotics on the Y-axis. Both isolates and antibiotics are ordered by hierarchical clustering, reflected by the horizontal and vertical trees, respectively. Antibiotic abbreviations are used as tabulated in A. Four clusters of interest (*w*, *x*, *y*, and *z*) representing different resistance patterns are annotated at the bottom of the heatmap. The heatmap was created in R by the basic *heatmap function*. **C**. A stacked bar plot summarizing the disc diffusion resistance patterns of the 198 *E. coli* isolates to 12 antibiotics (Y axis). Antibiotics are sorted by the same order in which they are clustered in B.

All isolates were screened for resistance against 12 antibiotics (Table S2) by the disc diffusion method. All isolates were resistant to penicillin and ampicillin. The next less effective antibiotic was the 2^nd^ generation cephalosporin, cefoxitin, to which 193 isolates (97.5%) appeared to be resistant. Regarding 3^rd^ generation cephalosporins, 142 isolates (71.7%) were resistant to cefotaxime and 153 isolates (77%) were resistant to ceftazidime, while 182 isolates (92%) were resistant to piperacillin and 105 isolates (53%) were resistant to piperacillin/tazobactam. Resistance to aztreonam, representing the monobactam class, was exhibited by 52% of the isolates. Finally, only five isolates (2.5%) were resistant to imipenem, representing the carbapenem class. This percentage of resistance was the lowest among all tested classes of beta-lactam antibiotics (Fig. 2A,C and Table S1).

**Figure 3:**
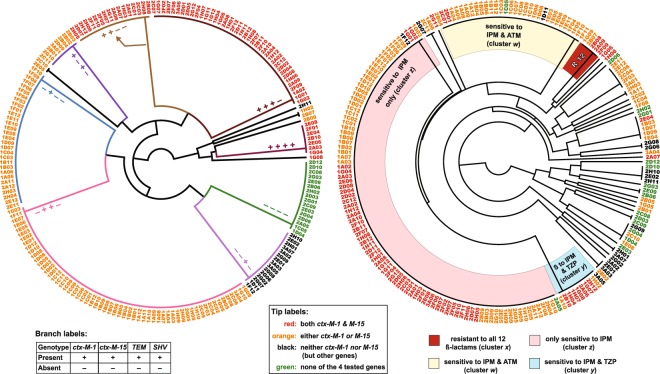
Trees showing relations between different isolates according to their beta-lactamase genotypes and antibiotic susceptibility phenotypes. Radial trees generated by the unweighted pair group method with arithmetic mean (UPGMA) method (http://genomes.urv.cat/UPGMA). Data were simplified as 1/0 values representing four resistance genotypes (left) and 12 resistant phenotypes (right). Isolate IDs (tree tips) are color-coded according to their *bla*_CTX-M_ genotype (as indicated in the figure key). Branches of the *genotype tree* (left) are labeled and colored according to their genotypes (+/−patterns), while four major clades in the *phenotype tree* (right) are colored according to their resistance pattern (representing clusters *w, x, y, z* in Fig. 2). The tree has the advantage of showing even the rarest phenotypes, e.g., that represented by isolate 1H06 (R to all antibiotics except AMC). The trees were drawn by FigTree and labeled in Adobe Illustrator CS5 as indicated in Materials and Methods.

To allow further analyses and comparisons, we defined a resistance score (R Score) as the number of beta lactams to which an isolate is resistant—out of 12 beta lactams tested. We also attempted to cluster all isolates based on their resistance pattern, and the clustering pattern was presented as a heatmap (Fig. 2B) and an unweighted pair group method with arithmetic mean (UPGMA) tree (Fig. 3). The heatmap has the advantage of showing the overall trends and patterns within the isolates, while the tree has the advantage of showing major clusters, as well as rare patterns (e.g., isolate 1H06, resistant to all antibiotics but AMC). Both methods highlighted some dominant clusters, most prominent of which are four that we named *w*, *x*, *y*, and *z* (Figs 2B, 3). Cluster *z* is the largest, representing isolates resistant to all antibiotics but imipenem, while Clusters *w* and *y* represent isolates resistant to all but IPM/ATM and IPM/TZP, respectively. Cluster x was not among the largest, but the most alarming, representing isolates resistant to all 12 beta-lactams.

### ESBL activity of the isolates

ESBL activity is usually expressed as synergism between the tested antibiotic and a mixture of amoxicillin/clavulanic acid. This is experimentally tested by disc diffusion and is typically exhibited as a keyhole (merged inhibition zone) formed between the tested antibiotic disc and a central amoxicillin/clavulanic acid disc on a typical surface-inoculated plate as detailed in Materials and Methods (Supplementary Fig. S1A).

When this test was applied to all 198 *E. coli* isolates, 134 showed positive synergism, indicative of their ESBL activity. Some isolates were resistant to the five antibiotics, which suggested the possibility that they expressed more than one ESBL gene.

A positive ESBL activity for a particular isolate is also expressed as an increase in the inhibitory zone diameter of either cefotaxime or ceftazidime by ≥5 mm when tested in combination with clavulanic acid *vs*. its zone diameter when tested alone (Supplementary Fig. S1B). When we used this method, 164 isolates showed positive results. This suggests that the combination disc method might be more sensitive than the double disc synergy (DDS) method.

### Factors affecting variability in resistance scores

**Figure 4:**
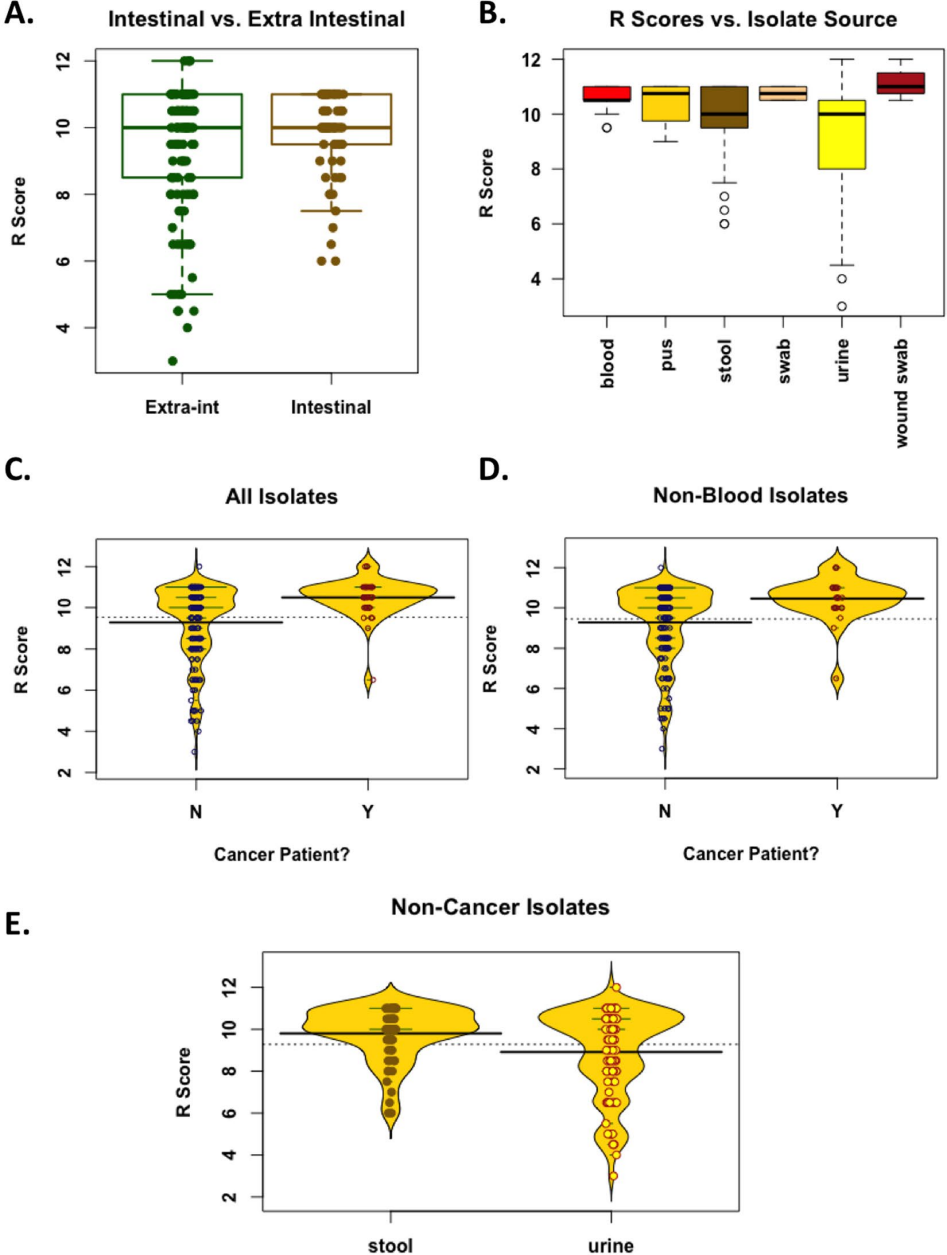
Phenotypic associations. (**A**) Boxplots showing the R scores of intestinal *vs*. extra-intestinal samples. Dots representing each sample were overlaid to show the actual variability and distribution of the data. Medians = 10,10; means = 9.35, 9.84, for extra-intestinal and intestinal isolates, respectively. The difference, however, was marginally significant, especially that the data distribution does not follow a normal distribution (t-test *p* value = 0.033; but Mann-Whitney *p* value = 0.44). (**B**) Boxplots showing R scores of isolates from different body sites (for any site from which more than three isolates were available). The means were generally significantly different (ANOVA F value = 4.908, *p* value = 0.003). (**C,D**) Beanplots comparing patients with cancer (Y) *vs*. non-cancer patients (N) according to the R scores of their isolates. Dots representing each sample were overlaid to show the actual variability and distribution of the data. (**C**) When all isolates are considered, the difference in mean R score = 1.22; t-test *p* value = 2.284 × 10^−8^; Mann-Whitney *p* value = 2.266 × 10^−5^. (**D**) When all but blood isolates are considered, the difference in mean R score = 1.18, t-test *p* value = 2.96 × 10^−5^; Mann-Whitney *p* value = 0.0009. (**E**) A beanplot with overlaid dots comparing the R scores of stool *vs*. urine isolates from non-cancer patients. Mann-Whitney W = 3632.5, *p* value = 0.00887. All plots were created by R basic functions or the *beanplot* package.

*E. coli’*s natural habitat is the intestine; however, in disease cases, the bacteria are frequently isolated from extra-intestinal sites. We thus investigated whether there is a difference in the overall resistance between intestinal and extra-intestinal isolates. Although the median R scores of both groups were equal (10), their means were slightly different (9.85 for intestinal isolates *vs*. 9.35 for extra-intestinal isolates). However, that difference was marginally significant (t-test *p* value = 0.033, but Mann-Whitney *p* value was 0.44, Fig. 4A).

Next, we tested whether the site of isolation or the nature of the disease might have an effect on the level of resistance. Overall, isolates from different body sites had more or less comparable R score values, except those from urine specimens (Fig. 4B). To statistically validate such observation, we filtered the data by excluding any body site from which fewer than three specimens were collected. Analysis of Variance (ANOVA) showed a significant difference between the means of all samples (*p* value = 0.003), but *post hoc* tests using FDR or Holm-Bonferroni adjustments indicated that the only significant pairwise differences were between the mean R scores of urine *vs*. blood and urine *vs*. stool isolates (FDR-adjusted *p* values = 0.0058 for both pairs; Holm-Bonferroni-adjusted *p* values = 0.01 for urine *vs*. blood and 0.012 for urine *vs*. stool). This significant difference between blood and urine (both being extra-intestinal) might explain the lack of statistical significance in resistance between all extra-intestinal isolates *vs*. the more consistent intestinal isolates.

As for disease associations, we observed a higher total resistance in isolates from cancer patients compared to those from patients with other conditions (Mean R score difference = 1.22; t-test *p* value = 2.284 × 10^−8^; Mann-Whitney *p* value = 2.266 × 10^−5^, Fig. 4C). However, because 15 out of 41 cancer patient isolates were from blood specimens whereas no blood specimens were obtained from non-cancer patients, it is possible that the higher resistance observed among these isolates is simply because they were from blood, possibly reflecting cases of secondary septicemia due to failed initial antibiotic therapy. Thus, we repeated the comparison between isolates from cancer and non-cancer patients after filtering out blood samples. Still, the differences between the R scores of these two classes of isolates remained significant (mean R score difference = 1.18, t-test *p* value = 2.96 × 10^−5^; Mann-Whitney *p* value = 0.0009, Fig. 4D).

The most prominent difference between isolates from cancer and non-cancer patients was manifested by their resistance to the most effective antibiotic in this study, imipenem (Table 1). Four out of five imipenem-resistant and two out of two imipenem-intermediate isolates were from cancer patients, indicating an enrichment of resistance to that last-resort antibiotic in cancer patients (Chi-square *p* value ≤ 0.0001), whereas the distribution of imipenem-sensitive samples was 35:156 (cancer to non-cancer patients, Table 1).

**Table 1:** Contingency table showing the distribution of Imipenem resistance phenotypes among cancer and non-cancer patients. Phenotype-phenotype associations are tested by Chi-square test for independence. %row = percentage of row total.

IPM resistance	Cancer	Non-cancer	Total
	N (%row)	N (%row)	N (%row)
R	4 (80%)	1 (20%)	5 (100%)
I	2 (100%)	0 (0%)	2 (100%)
S	35 (18.3%)	156 (81.7%)	191 (100%)
Total	41 (20.7%)	157 (79.3%)	198 (100%)

Chi-square = 19.02 with 2 df p ≤ 0.0001.

Regarding patterns of resistance to specific antibiotics, we found that aztreonam resistance was enriched in isolates from blood and pus (100%), while the percentage of aztreonam-resistant isolates in urine and stool was 50%. Likewise, cefotaxime resistance was predominant in isolates from blood and pus (100%), but, in urine and stool, the percentage of resistant isolates was 50%.

Two more antibiotics (TZP and CAZ) had substantial resistance in most isolates, but with a significantly smaller proportion of resistant bacteria isolated from urine specimens, a significantly larger proportion of TZP-resistant bacteria from stool, and a significantly larger proportion of CAZ-resistant bacteria from blood and pus (Chi-square *p* values = 0.0001 and 0.0003, respectively, Table 2).

**Table 2:** Contingency tables showing the distribution of resistance to selected antibiotics according to the site of isolation.

TZP resistance	Blood	Pus swab	Stool	Urine	Total
	N (%col)	N (%col)	N (%col)	N (%col)	N (%col)
R	10 (66.7%)	6 (60%)	52 (73.2%)	34 (35.1%)	102 (52.9%)
I	2 (13.3%)	3 (30%)	9 (12.7%)	38 (39.2%)	52 (26.9%)
S	3 (20%)	1 (10%)	10 (14.1%)	25 (25.8%)	39 (20.2%)
Total	15 (100%)	10 (100%)	71 (100%)	97 (100%)	193* (100%)
Chi-square = 27.17 with 6 df; p = 0.0001					
**CAZ resistance**	**Blood**	**Pus swab**	**Stool**	**Urine**	**Total**
	**N (%col)**	**N (%col)**	**N (%col)**	**N (%col)**	**N (%col)**
R	15 (100%)	10 (100%)	63 (88.7%)	60 (61.9%)	148 (76.7%)
I	0 (0%)	0 (0%)	2 (2.82%)	13 (13.4%)	15 (7.77%)
S	0 (0%)	0 (0%)	6 (8.45%)	24 (24.7%)	30 (15.5%)
Total	15 (100%)	10 (100%)	71 (100%)	97 (100%)	193* (100%)
Chi-square = 25.50 with 6 df; p = 0.0003					

Distribution of piperacillin/tazobactam (TZP) and ceftazidime (CAZ) resistance phenotypes according to site of isolation. Phenotype-phenotype associations are tested by Chi-square test for independence. %col = percentage of column total.

*The total N = 193 after exclusion of tissues from which two or fewer samples were isolated.

It is to be noted that we excluded in this statistical analysis any tissues from which two or fewer samples were isolated.

### Antibiotic-antibiotic correlations

**Figure 5:**
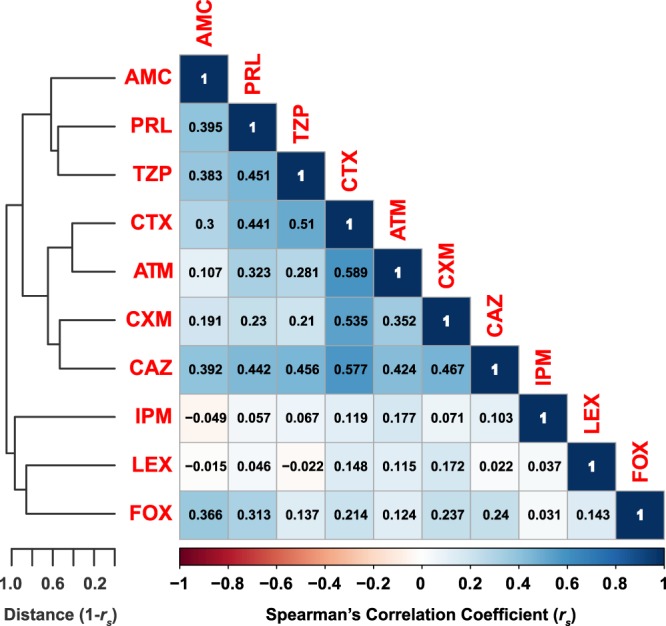
Antibiotic-antibiotic correlations. A pseudo-colored asymmetric correlation matrix representing the correlations between each pair of antibiotics according to the patterns of susceptibility of all 198 isolates. Penicillin and ampicillin were not included as all isolates were resistant to them. Spearman’s rank correlation coefficients (r_s_) are shown for each antibiotoic pairs. The antibiotics are hierarchically clustered, and the clustering is represented by the tree shown to the left. Tree branches represent 1-r_s_. The *CorrPlot* R package and the basic *hclust* R function were used to create this figure.

It is logically expected that bacteria may have similar susceptibility patterns to antibiotics belonging to closely related chemical classes. To test this hypothesis, we used the resistance data (Table S1) from all 198 samples to build a correlation matrix between different tested antibiotics (Fig. 5), with exclusion of penicillin and ampicillin, to which all isolates were resistant—with no exception.

This analysis indicated that cefotaxime (CTX), aztreonam (ATM), cefuroxime (CXM), and ceftazidime (CAZ) were the most correlated. For instance, the Spearman’s correlation coefficient *r*_*s*_ between CTX and ATM was 0.589; between CTX and CAZ was 0.577; between CTX and CXM was 0.535; finally, CTX and TZP had a correlation coefficient = 0.510. All other Spearman’s correlation coefficients were ≤0.5 (Fig. 5).

### Polymerase chain reaction (PCR) screening of beta-lactamase genes and genotype-phenotype correlation

For the determination of beta-lactamase genotype among the isolates, one-product and multiplex PCR assays were used to detect representative genes (as detailed in Materials and Methods). Different patterns of gene presence/absence were observed (Supplementary Table S1), and these patterns were used to generate a UPGMA tree leading to different clusters, the largest of which represents isolates positive for *bla*_CTX-M-15_ and *bla*_TEM_ genes (Fig. 3).

Next, the isolates were screened by universal CTX primers (MA1/MA2 primers^11^) to detect the presence of any *bla*_CTX-M_ genes, then a multiplex PCR was performed to show which CTX-M group is present in each isolate. Afterward, primers for the most predominant members of each CTX-M group were designed and used for further screening (Supplementary Table S3).

One hundred seventy nine isolates were successfully amplified with the universal PCR primers (MA1 and MA2)^11^, which indicates that they contain at least one of the *bla*_CTX-M_ genes. Those MA1/MA2-positive isolates were screened by a multiplex PCR to determine to which of the five CTX-M groups (1, 2, 8, 9, or 25) their *bla*_CTX-M_ genes belonged; then the samples that tested positive for CTX-M-group 1 genes were further screened by two CTX primers (CTX-M-1 and CTX-M-15, the most predominant *bla*_CTX-M_).

Intriguingly, out of the 179 MA1/MA2-positive isolates, 155 (86.6%) isolates tested positive when amplified with CTX-M-15 primers while only 75 (41.9%) tested positive with CTX-M-1 primers. The results suggest that *bla*_CTX-M-15_ is the most predominant in the isolates; still, the two primer pairs (CTX-M-1 and CTX-M-15), used together, seem to provide better prediction of overall resistance as well as resistance to specific beta lactams.

**Figure 6:**
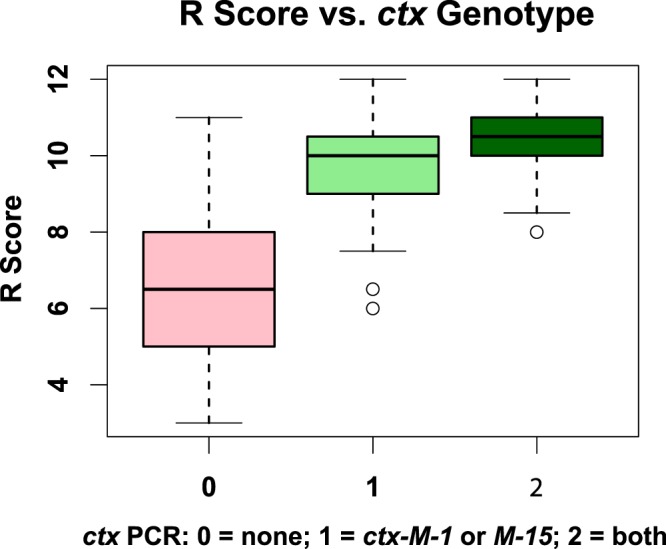
Genotype-phenotype associations. Boxplots showing the relation between R score of an isolate and the detection of *bla*_CTX-M-1_ or *bla*_CTX-M-15_ or both by PCR. The three groups were statistically significantly different (ANOVA *p* value = 2 × 10^−16^). *Post hoc* one-tailed t tests with Bonferroni adjustment indicated that mean R scores are still significantly higher with the detection of both genes than with the detection of one gene (adjusted *p* value = 0.00052). Expectedly, the difference was more statistically significant with the absence of both genes (adjusted *p* value = 2 × 10^−16^).

Regarding overall resistance, the R score was found to be significantly higher in isolates in which either *bla*_CTX-M-1_ or *bla*_CTX-M-15_ was detected by PCR (Fig. 6). More interestingly, isolates that tested positive for both *bla*_CTX-M-1_ and *bla*_CTX-M-15_ had a significantly higher mean R score than those with a positive PCR result for only one of the two *bla*_CTX-M_ genes (*p* = 0.0005) or for neither (*p* = 2 × 10^−16^). This suggests that using both primer pairs might have greater diagnostic value.

Likewise, both primer pairs seemed to be needed for the best prediction of aztreonam (ATM), cefotaxime (CTX), and ceftazidime (CAZ) resistance (Table 3). For example, 62.2% of the ATM-sensitive isolates tested positive with only one of the primer pairs. Conversely, in the cases where PCR was positive with both primer pairs, only three isolates were still sensitive in spite of the presence of both genes (Table 3).

**Table 3:** Contingency tables linking some resistance phenotypes (to ATM, CTX and CAZ) to *bla*_CTX-M_ PCR results (for *bla*_CTX-M-1_ and *bla*_CTX-M-15_).

*bla*_*CTX-M*_ PCR	Both negative	Either positive	Both positive	Total
ATM resistance	N (%row)	Either positive	N (%row)	
R	3 (2.91%)	41 (39.8%)	59 (57.3%)	103 (100%)
I	4 (19.0%)	15 (71.4%)	2 (9.52%)	21 (100%)
S	25 (33.8%)	46 (62.2%)	3 (4.05%)	74 (100%)
Total	32 (16.2%)	102 (51.5%)	64 (32.3%)	**198 (100%)**
***bla***_***CTX-M***_ **PCR**	**Both negative**	**Either positive**	**Both positive**	**Total**
**CTX resistance**	**N (%row)**	**N (%row)**	**N (%row)**	
R	7 (4.93%)	74 (52.1%)	61 (43.0%)	142 (100%)
I	7 (30.4%)	13 (56.5%)	3 (13.0%)	23 (100%)
S	18 (54.5%)	15 (45.5%)	0 (0%)	33 (100%)
Total	32 (16.2%)	102 (51.5%)	64 (32.3%)	**198 (100%)**
***bla***_***CTX-M***_ **PCR**	**Both negative**	**Either positive**	**Both positive**	**Total**
**CAZ resistance**	**N (%row)**	**N (%row)**	**N (%row)**	
R	8 (5.23%)	85 (55.6%)	60 (39.2%)	153 (100%)
I	1 (6.67%)	10 (66.7%)	4 (26.7%)	15 (100%)
S	23 (76.7%)	7 (23.3%)	0 (0%)	30 (100%)
Total	32 (16.2%)	102 (51.5%)	64 (32.3%)	**198 (100%)**
Chi-square for ATM resistance vs. PCR results = 73.02 with 4 df; p ≤ 0.0001				
Chi-square for CTX resistance vs. PCR results = 62.70 with 4 df; p ≤ 0.0001				
Chi-square for CAZ resistance vs. PCR results = 97.98 with 4 df; p ≤ 0.0001				

Phenotype-genotype associations are tested by Chi-square test for independence. % row = percentage of row total.

With cefotaxime resistance, the results were even more striking, as none of the 64 isolates that tested positive with both PCR primer pairs was actually sensitive to CTX. While 18 of the 33 CTX-sensitive isolates tested negative for both, 15 of these 33 isolates had one of the two genes (Table 3). Specifically, among the 15 sensitive isolates that tested positive to one gene, 13 (86.7%) were *bla*_CTX-M-15_ positive and the remaining two were *bla*_CTX-M-1_ positive. This isn’t too surprising given the predominance of the M-15 alleles among our samples.

Resistance to CAZ followed a quite similar pattern to that of CTX in relation to PCR results. This, too, could be expected from the relatively high positive correlation between the two antibiotics (*r*_*s*_ = 0.577, Fig. 5).

Universal primers for other beta-lactamases genes (TEM and SHV) were also used for beta-lactamase genotyping or gene screening. Only 132 isolates generated positive TEM PCR products, while 12 isolates were SHV-positive (Table S1). Of note, isolates containing more than one type of *bla* genes (e.g., *bla*_*TEM*_ and *bla*_CTX-M_) showed more phenotypic resistance than those containing only one *bla* gene (data not shown).

### *Bla*_CTX-M_ genotypes and the discovery of two novel alleles

Finally, we successfully sequenced 88 out of the 179 *bla*_CTX-M_ PCR-positive isolates. The vast majority of the sequences were identical to the *bla*_CTX-M-15_ sequence (82 samples); three matched *bla*_CTX-M-14_; two matched *bla*_CTX-M-1_; and one was closest to *bla*_CTX-M-3_ (Table S1).

Among the *bla*_CTX-M-15_ and *bla*_CTX-M-14_ like genes amplified in this study, we discovered and confirmed two novel alleles (Supplementary Fig. S2), which we named *bla*_CTX-M-15.2_ and *bla*_CTX-M-14.2_, respectively. Both novel alleles were confirmed by resequencing and deposited in NCBI (Accession numbers: KX013145.1 and KX013146.1, respectively).

## Discussion

*Escherichia coli* is a natural inhabitant of the mammalian intestine, but is also recognized as one of the main causes of nosocomial, intestinal and extra-intestinal infections^12^, and a leading cause of morbidity and mortality over the world. According to the World Health Organization (WHO) 2015 report, *E. coli* causes disease to about 550 million people annually, of whom 230,000 eventually die^13^.

More importantly and worrisomely, *E. coli* is among the most likely bacteria to evolve into incurable *superbugs* in 2050, by developing resistance to every known antibiotic, posing a public health threat that is expected to exceed cancer’s mortality^2^. Indeed, recent reports of colistin-resistant *E. coli*^14–17^ raised alarm around the globe and ushered in the start of an era of pan-resistant bacteria.

Antimicrobial resistance in *E. coli* has gained much attention since it is the best studied Gram-negative organism in humans. Cases of developed resistance against beta-lactam antibiotics and others are increasingly documented, and several studies reported the increase of *E. coli* resistance to more than one drug or class of drugs^18^. Surveillance data show that resistance of *E. coli* is higher when antimicrobial agents have been used for longer durations, especially beta-lactam antibiotics^18^. This continuous exposure to antibiotics, which exerts selection on the bacteria, is boosted by the bacterial ability to acquire resistance factors from its neighbors (via horizontal gene transfer), thus allowing the emergence of strain variants that carry diverse resistance traits.

In agreement with global reports, an alarming increase in resistance to beta-lactam antibiotics (even to the extended-spectrum subclass) among clinical *E. coli* isolates is highlighted by the results of this study. Among 198 multi-resistant clinical isolates, 179 (90.4%) contained at least one *bla*_CTX-M_ gene, of which 86.6% were positive for *bla*_CTX-M-15_. A possible reason behind those high percentages is that many clinical isolates were recovered from the National Cancer Institute, in which high amounts of antibiotics are administered to patients pre- and post-operatively. Yet, even with exclusion of isolates from cancer patients, the percentage and distribution of resistance genes didn’t change much, as detailed in the Results section (Fig. 4E).

This high frequency of finding *bla*_CTX-M-15_–containing isolates agrees with previous reports on *bla*_CTX-M-15_ being the most common ESBL in the Middle East and North Africa^19^. This increasing predominance of the *bla*_CTX-M-15_ allele might be due to the powerful ability of its gene products (Ctx-M-15 and its variants) to hydrolyze ceftazidime, cefotaxime and aztreonam, which probably offers the bacteria a selective advantage especially when multiple antibiotics are concomitantly or consecutively prescribed.

Among 12 antibiotics screened in this study, only imipenem may be considered to have remained resilient; however, it will not be long until extended exposure to imipenem will have its impact on selecting for bacteria that are resistant to this last-resort antibiotic. Recent reports have already described the emergence of carbapenem resistance in Enterobacteriaceae^20^.

Of note, the pairwise correlation between bacterial resistance patterns to all antibiotics tested in this study was explored. Since all isolates were resistant to penicillin and ampicillin, it was not possible to test any correlations with these two relatively old antibiotics. On the other hand, a significant but partial pairwise correlation was found between the four antibiotics: CTX, ATM, CXM, and CAZ. This partial correlation reflects possible common mechanisms of resistance to these antibiotics, yet highlights that there are still discrepancies between how bacteria resist those beta lactams. Discrepancies could be due to slight differences in resistance mechanisms, differences in enzyme specificities, or potential chemical differences.

Associations were also explored between levels and patterns of resistance to different antibiotics and some clinical aspects, such as the site of isolation or the disease condition (cancer or non-cancer). When levels of resistance of bacteria from various sites of isolation were compared, there was no striking difference in R Scores, except with urine isolates, which had slightly but significantly lower R Scores. Yet, these urine isolates had remarkably the highest variance between their R scores (Fig. 4B,E).

When cancer was tested as a possible factor for higher risk of multiple resistance phenotypes, it was found as a significant predictor of R Score; however, this statistically significant difference is diluted by the difference of sampling sites, as many of the cancer patient isolates were recovered from blood and other tissues, while the majority of other isolates were from stool and urine. When non-cancer isolates were compared, the same pattern of lower resistance among urine isolates was observed.

It is still plausible to suggest a tendency of higher resistance in bacteria isolated from cancer patients, as they often suffer from chemotherapy-induced immunosuppression, which necessitates the use of multiple antibiotics in their therapy regimens. An example in this study is the detection of rare resistance to imipenem in only five isolates, four of which were from patients with cancer. This disproportionate distribution suggests that resistance to imipenem started developing in cancer patients, but not in other patients who were sampled in the study. On the other hand, as opposed to the effect of ‘having cancer’ as a predictor, we think there is not enough sample size or statistical power to fully explain the higher variance observed in urine samples. The major factor here seems to be whether the patient has cancer or not.

After phenotypic determination of susceptibility and resistance phenotypes, we resorted to molecular tools to explore the distribution of representative beta-lactamase genes, with focus on *bla*_CTX-M_ genes and their variants. We found that *bla*_CTX-M_ containing isolates were resistant to a wider range of beta-lactam antibiotics than those not containing a *bla*_CTX-M_ gene (Table 3). This could be an indication that the presence of a *bla*_CTX-M_ gene in a bacterium might be leveraged as a good biomarker for high resistance to beta-lactam antibiotics, and ought to be implemented in routine antibiotic susceptibility testing protocols.

A major goal of this study was to explore the extent of agreement/correlation between phenotypic and molecular assays (i.e., resistance phenotype–genotype correlation). For this purpose, more than one phenotype can be measured and considered. For example, phenotypic resistance to single antibiotics may be used as an indicator, especially third- and fourth- generation antibiotics, or those considered as last resort, such as imipenem. Another phenotypic indicator is the breadth of resistance to beta-lactams or the level of multiresistance to this class (expressed here as *R score*). Analyzing phenotype–genotype correlations is especially beneficial in the case of secondary mutations, which are sometimes overlooked, although they often play an important role in potentiating or attenuating resistance phenotypes.

A significant association between overall resistance to beta lactams and molecular tests was found with the use of a single CTX-M primer pair, but the use of two primer pairs (targeting *bla*_CTX-M-1_ and *bla*_CTX-M-15_) had unexpectedly higher predictive value of the overall resistance scores (R scores, Fig. 6).

This observation is bolstered by parallel genotype-phenotype clustering analysis (Fig. 3), which provides a good summary of the study and shows a partial but obvious phenotype-genotype agreement. Although the phenotype and genotype UPGMA trees (Fig. 3) do not fully superimpose, it is visually striking that most isolates positive to two *bla*_CTX-M_ genes are within the highly resistant clusters (clusters *x* and *z*, Figs 2B, 3). On the other hand, only one isolate that had none of the screened resistance genes, 2A05 (labeled in green, Fig. 3), had a highly resistant phenotype (R score = 10.5 and part of Cluster *z*, Fig. 3).

Finally, perhaps the most impactful finding of this study, other than the genotype-phenotype correlations, is the determination of novel *bla*_CTX-M_ gene variants, in spite of the vast predominance of *bla*_CTX-M-15_ among isolates. This discovery of two novel but rare alleles highlights the value of using a relatively large number of isolates, as the dominance of one *bla*_CTX-M_ genotype (M-15 as reported here and in several studies from the region) may mask other novel variants. Thus using a large number of isolates (here, *bla*_CTX-M_ was screened in 198 isolates and its gene was sequenced from 88 of them) is a key to finding novel variants, which might eventually spread and be the next dominant genotypes. The two novel alleles were named *bla*_CTX-M-14.2_ and *bla*_CTX-M-15.2_. To avoid muddling the *bla*_CTX-M_ nomenclature, we refrained from using new serial numbers until further phenotypic studies are conducted on whether the sequence variations offer any difference in the MIC, or in the enzymatic activity and enzyme kinetics.

In conclusion, using antibiotic sensitivity data for a couple of hundred clinical *E*. coli isolates, we were able to investigate a few clinical and molecular variables for their diagnostic value, and we discovered novel beta lactamase alleles. Additionally, we explored phenotype–phenotype and phenotype–genotype correlations within and between the isolates and the tested antibiotics. Future studies with larger data sets may be able to build networks and models to move from diagnosis to prediction of resistance emergence and hopefully interference with its pervasive dissemination.

## Materials and Methods

### Ethical statement

All experiments and study protocols were in accordance with relevant guidelines, regulations, and international declarations for biomedical research ethics. All protocols, including sampling, handling biobanked samples, and analyzing anonymized patient records, were approved by the Ethics Committee of Faculty of Pharmacy, Cairo University (Approval# 923; year 2012 to AAR and MAA). Informed consent was obtained from all hospitalized patients or their legal guardians according to each hospital’s ethical protocols. The authors had no access to any patient-identifying data, as the samples were obtained from hospital laboratories with anonymized copies of patient data that are only related to diagnosis, site of infection, and underlying diseases.

### Microorganisms

Two hundred and fifty bacterial isolates were obtained from biobanked stocks in clinical laboratories of Qasr El-Ainy Educational Hospital, Abu-El-Rish Children Hospital, and the National Cancer Institute in Cairo-Egypt in the period between January and April 2013. Isolates were identified by conventional microbiological methods to the genus level^21^. Complete identification of the recovered isolates was carried out by biochemical reactions including oxidase test, citrate utilization, growth on Triple sugar iron (TSI), H_2_S production, and Indole Methyl Red Vogues Proskauer Citrate (IMViC) test, as well as API system (bioMérieux, Lyon, France).

### Determination of antimicrobial susceptibility pattern by disc diffusion method and calculation of a *Resistance Score*

The sensitivity of the tested isolates against 12 antibiotics, representing different classes of beta-lactams (Supplementary Table S2), was determined by the disc diffusion method according to the Clinical and Laboratory Standards Institute (CLSI) guidelines^22–24^.

A *resistance score* (R Score) was defined for each isolate. The score was defined as the number of antibiotics to which this isolate is resistant. If an isolate was judged as intermediately resistant to an antibiotic, it was given a score of 0.5, while any *resistant* call was given a score of 1.

### ESBL confirmatory tests using disc diffusion

#### Double disc synergy test (DDST)

The Double disc synergy test (DDST) used in this study was modified from Jarlier’s double-disc synergy (DDS) method^25^. Discs of cefotaxime, cefepime, ceftazidime, and aztreonam were placed around an amoxicillin/clavulanic acid disc at a distance of 20 mm (center to center^25,26^). Positive ESBL production was marked by the observation of a keyhole (Supplementary Fig. S1A).

#### Combination Disc test

This confirmatory test, recommended by CLSI^24^, requires the use of ceftazidime and cefotaxime, with and without clavulanic acid. Discs used were ceftazidime (30 µg), ceftazidime-clavulanic acid (30/10 µg), cefotaxime (30 µg), and cefotaxime-clavulanic acid (30/10 µg). If the zone diameter increased by ≥ 5 mm after either antibiotic was combined with clavulanic acid, this was taken as evidence for the presence of an ESBL production^27,28^ (Supplementary Fig. S1B).

### Molecular identification of ESBL genotype using PCR

PCR was used to detect the presence of antibiotic resistance genes in *E. coli* showing phenotypic resistance to beta lactamases. Bacterial colonies were boiled in TE buffer for 5 min, flash centrifuged, and then the supernatant of boiled colonies was used as a PCR template. Go Taq DNA polymerase enzyme (Promega, Madison, WI, USA) was used for all amplifications. Primers are shown in Supplementary Table S3.

Amplification conditions for all reactions were: initial Denaturation at 94 °C for 5 min; 30 cycles of denaturation at 94 °C for 1 min, annealing at 52 °C for 45 s, and elongation at 72 °C for 1 min; then a final elongation at 72 °C for 10 min. These conditions were applied to all one-product or multiplex PCR reactions except for the following primer pairs: TEM, SHV, CTX-M-1, CTX-M-2, and CTX-M-15 which were annealed at 56 °C, 43 °C, 46 °C, 50 °C, and 50 °C, respectively.

### Nucleotide sequence of amplified *bla*_CTX-M_ genes

To confirm the identity of *bla*_CTX-M_ genes detected by PCR, and to determine any possible new *bla*_CTX-M_ alleles specific to this study, we extracted the DNA out of the electrophoretic bands representing positive *bla*_CTX-M_ PCR products (using QIAGen Gel purification kit, QIAGen, Germantown, MD, USA). This DNA was afterward sequenced by the Sanger method (Clinilab, Cairo, Egypt and EtonBio, San Diego, CA, USA) with the MA1 and MA2 primers^11^.

All obtained sequences were visually revised and corrected for minor electropherogram errors, then were compared to sequence databases by BlastN and BlastX with default settings and parameters^29^.

### Statistical analysis

Several programs and software packages were used for data visualization, graphing, summary statistics, and hypothesis testing: these are the R statistical platform (https://www.r-project.org), Data Desk version 6.3 (Ithaca, NY, USA), and GraphPad Prism (La Jolla, CA, USA). Among R-packages used in data analysis and visualization are Readxl (version 1.0), Beanplot (version 1.2), and Corrplot (version 0.84). UPGMA clustering (http://genomes.urv.cat/UPGMA) was used for generating a distance matrix and a tree, which was drawn with FigTree (http://tree.bio.ed.ac.uk/software/figtree/). Graphs and trees were sometimes labeled or recolored on Adobe Illustrator CS5 version 15.0.0 (Adobe Systems, Inc., San Jose, CA, USA).

## Supplementary information

Supplementary Information

Table S1

## Data Availability

All data generated or analyzed during this study are included in the published article and its supplementary information files. New Sequences identified in this study have been deposited in NCBI GenBank with the accession numbers KX013145.1 and KX013146.1 (http://www.ncbi.nlm.nih.gov).
