# Supplementary material for: Novel blaCTX-M variants and genotype-phenotype correlations among clinical isolates of extended spectrum beta lactamase-producing Escherichia coli
Source: Sci Rep. 2019 Mar 12;9:4224. doi: 10.1038/s41598-019-39730-0 (PMC6414621; doi:10.1038/s41598-019-39730-0)
Supplement: Supplementary file 1 — Supplementary Information [file 41598_2019_39730_MOESM1_ESM.pdf]

**Novel *ctx* Variants and Genotype-Phenotype Correlations among Clinical Isolates of Extended Spectrum Beta Lactamase-producing *Escherichia coli***

**Ahmed A. Ramadan[1], Neveen A. Abdelaziz[1], Magdy A. Amin[2], Ramy K. Aziz[2,3*]**
[1]Department of Microbiology and Immunology, Faculty of Pharmacy, Ahram Canadian University, Giza, Egypt; [2]Department of Microbiology and Immunology, Faculty of Pharmacy, Cairo University, Cairo, Egypt; [3]Center for Microbiome and Genome Research, Cairo University, Cairo, Egypt

**Running Title:** Genotype-phenotype correlations among ESBL *E. coli*

* To whom correspondence should be addressed
Address: Department of Microbiology and Immunology, Faculty of Pharmacy, Cairo University, Qasr El-Ainy Street, 11562 Cairo, Egypt

**CONTENTS:**

**Supplementary Tables:**

**Table S1** All raw data related to the isolates, their sources, their sensitivity to all tested antibiotics, and their PCR results (online file)

**Table S2** List of discs used for antimicrobial susceptibility testing

**Table S3** PCR primers for screening beta lactamase genes or determining the CTX genotypic group

**Supplementary Figures:**

**Figure S1** Representative *in vitro* tests used to determine the ESBL phenotypes

**Figure S2** BlastN and BlastX alignment of the newly identified *ctx* alleles to their best-matching genes

**Table S2: List of discs used for antimicrobial susceptibility testing**

| **Antibiotic name** | **Abbre-viation** | **Disc Content ($\mu$g)** |
|---|---|---|
| Amoxicillin/ clavulanic acid | AMC | 30 |
| Ampicillin | AMP | 10 |
| Aztreonam | ATM | 30 |
| Cefalexin | LEX | 30 |
| Cefotaxime | CTX | 30 |
| Ceftazidime | CAZ | 30 |
| Cefuroxime | CXM | 30 |
| Cefoxitin | FOX | 30 |
| Imipenem | IPM | 10 |
| Penicillin G | P | 10 |
| Piperacillin | PRL | 100 |
| Piperacillin/ Tazobactam | TZP | 110 |

**Table S3: PCR primers for screening beta lactamase genes or determining the CTX genotypic group.**
(Prod. = product; F = forward amplification primer; R = reverse amplification primer)

| Target | Oligonucleotide sequence (5' to 3') | Prod. size | Purpose | Source |
|---|---|---|---|---|
| ***bla TEM*** | **F:** TCGTGTCGCCCTTATTCCCTT<br>**R:** GCGGTTAGCTCCTCCGGTCCT | 409 | PCR screening for beta-lactamase type | Ref 1 |
| ***bla SHV*** | **F:** CGGCCCCGCAGGATTGACT<br>**R:** TCCCGGCGATTTGCTGATTTC | 391 | PCR screening for beta-lactamase type | Ref 1 |
| ***bla CTX-M-1*** | **F:** GTTAATTCGTCTCTTCCAGAATAAGG<br>**R:** GCTTATTCATCGCCACGTTATC | 453 | PCR screening for beta-lactamase type | This study |
| ***bla CTX-M-2*** | **F:** CGATGTGCAGTACCAGTAA<br>**R:** TTAGTGACCAGAATCAGCGG | 585 | PCR screening for beta-lactamase type | Ref 2 |
| ***bla CTX-M-9*** | **F:** GAGAGTGCAACGGATGATGT<br>**R:** CGGTATTCAGCGTAGGTTCAG | 512 | PCR screening for beta-lactamase type | This study |
| ***bla CTX-M-15*** | **F:** GCGCTACAGTACAGCGATAA<br>**R:** TTTACCCAGCGTCAGATTCC | 428 | PCR screening for beta-lactamase type | This study |
| ***bla CTX-M-25*** | **F:** CTGGGTGTGGCGTTGATTA<br>**R:** TCGTGCCATTGACGTGTT | 213 | PCR screening for beta-lactamase type | This study |
| **Group 1 CTX** | **F:** AAAAATCACTGCGCCAGTTC<br>**R:** AGCTTATTCATCGCCACGTT | 415 | Multipex PCR for CTX genotyping | Ref 3 cited in 4 |
| **Group 2 CTX** | **F:** CGACGCTACCCCTGCTATT<br>**R:** CCAGCGTCAGATTTTTCAGG | 552 | Multipex PCR for CTX genotyping | Ref 3 cited in 4 |
| **Group 8 CTX** | **F:** TCGCGTTAAGCGGATGATGC<br>**R:** AACCCACGATGTGGGTAGC | 666 | Multipex PCR for CTX genotyping | Ref 3 cited in 4 |
| **Group 9 CTX** | **F:** CAAAGAGAGTGCAACGGATG<br>**R:** ATTGGAAAGCGTTCATCACC | 205 | Multipex PCR for CTX genotyping | Ref 3 cited in 4 |
| **Group 25 CTX** | **F:** GCACGATGACATTCGGG<br>**R:** AACCCACGATGTGGGTAGC | 327 | Multipex PCR for CTX genotyping | Ref 3 cited in 4 |
| ***ctx–M*** **Universal primers** | **MA1:** SCSATGTGCAGYACCAGTAA<br>**MA2:** CCGCRATATGRTTGGTGGTG | variable | Amplifying all known *ctx* alleles | Ref 5 |

**Figure S1.**

**A.** **B.**

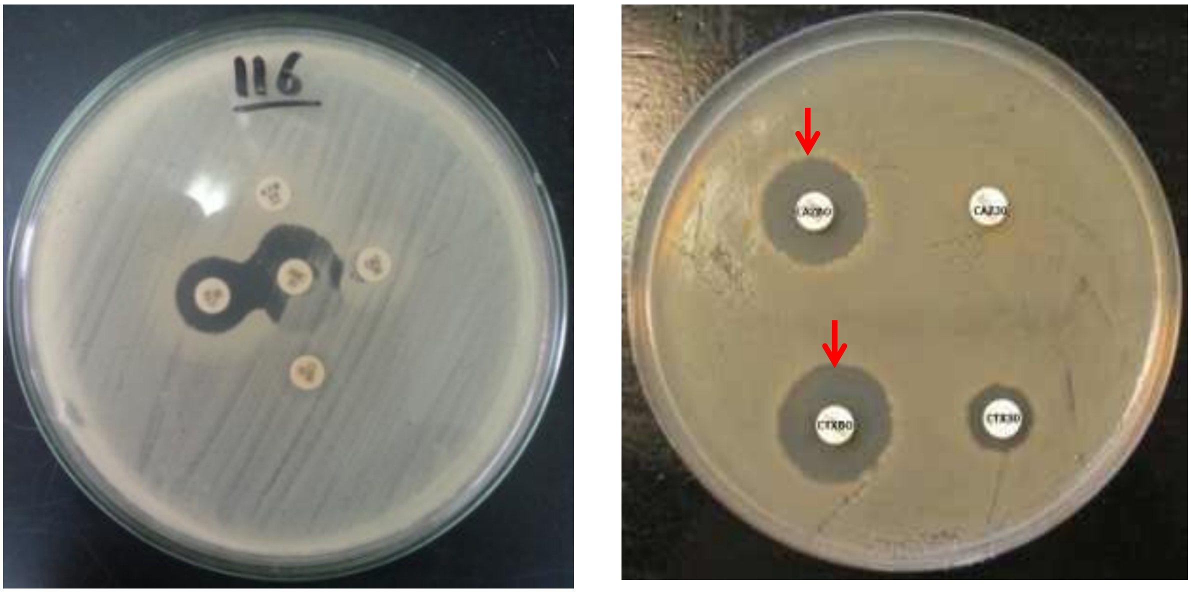


**Figure S1. Representative *in vitro* tests used to determine the ESBL phenotypes**

**A.** The typical keyhole pattern that indicates a positive ESBL isolate in the DDST. **B.** A representative positive result of the combined disc method: the zone diameter increased around the discs to which clavulanic acid was added (indicated with arrows)

## FIGURE S2

### A. BlastN best hit of *ctx-M-14.2*

```
Query   1    CAGCTCGGTGGCCCGGGAGGCGTGACGGCTTTTGCCCGCGCGATCGGCGATGAGACGTTT  60
             ||||||||||||||||||||||||||||||||||||||||||||||||||||||||||||
Sbjct   386  CAGCTCGGTGGCCCGGGAGGCGTGACGGCTTTTGCCCGCGCGATCGGCGATGAGACGTTT  327

Query   61   CGTCTGGATCGCACTGAACCTACGCTGAATACCGCCATTCCCGGCGACCCGAGAGACACC  120
             ||||||||||||||||||||||||||||||||||||||||||||||||||||||||||||
Sbjct   326  CGTCTGGATCGCACTGAACCTACGCTGAATACCGCCATTCCCGGCGACCCGAGAGACACC  267

Query   121  ACCACGCCGCGGGCGATGGCACAGACGTTGCGTCAGCTTACGCTGGGTCATGCGCTGGGC  180
             ||||||||||||||||||||||||||||||||||||||||||||||||||||||||||||
Sbjct   266  ACCACGCCGCGGGCGATGGCACAGACGTTGCGTCAGCTTACGCTGGGTCATGCGCTGGGC  207

Query   181  GAAACCCAGCGGGCGCAGTTGGTGACGTGGCTCAAAGGCAATACGACCGGCGCAGCCAGC  240
             ||||||||||||||||||||||||||||||||||||||||||||||||||||||||||||
Sbjct   206  GAAACCCAGCGGGCGCAGTTGGTGACGTGGCTCAAAGGCAATACGACCGGCGCAGCCAGC  147

Query   241  ATTCGGGCCGGCTTACCGACGTCGTGGACTGTAGGTGATAAGACCGGCAGCGGCGACTAC  300
             ||||||||||||||||||||||||||||||||||||||||||||||||||||||||||||
Sbjct   146  ATTCGGGCCGGCTTACCGACGTCGTGGACTGTAGGTGATAAGACCGGCAGCGGCGACTAC  87

Query   301  GGCACCACCAACCATATCGCGG  322
             |||||||||||  |||| ||||
Sbjct    86  GGCACCACCAATGATATTGCGG  65
```

### B. BlastX best hit of *ctx-M-14.2*

```
Query   1    QLGGPGGVTAFARAIGDETFRLDRTEPTLNTAIPGDPRDTTTPRAMAQTLRQLTLGHALG  180
             QLGGPGGVTAFARAIGDETFRLDRTEPTLNTAIPGDPRDTTTPRAMAQTLRQLTLGHALG
Sbjct   72   QLGGPGGVTAFARAIGDETFRLDRTEPTLNTAIPGDPRDTTTPRAMAQTLRQLTLGHALG  131

Query   181  ETQRAQLVTWLKGNTTGAASIRAGLPTSWTVGDKTGSGDYGTTNHIA  321
             ETQRAQLVTWLKGNTTGAASIRAGLPTSWTVGDKTGSGDYGTTN IA
Sbjct   132  ETQRAQLVTWLKGNTTGAASIRAGLPTSWTVGDKTGSGDYGTTNIIA  178
```

## C. BlastN best hit of *ctx-M-15.2*

```
Query     1  GCGGTGCTGCTGAGAAGTGAAAGCGAACCGAATCTGTTAAATCAGCGAGTTGAGATCAAA  60
             |||||||||  || ||||||||||||||||||||||||||||||||||||||||||||||
Sbjct  2593  GCGGTGCTGAAGAAAAGTGAAAGCGAACCGAATCTGTTAAATCAGCGAGTTGAGATCAAA  2652

Query  61    AAATCTGACCTTGTTAACTATAATCCGATTGCGGAAAAGCACGTCAATGGGACGATGTCA  120
             ||||||||||||||||||||||||||||||||||||||||||||||||||||||||||||
Sbjct  2653  AAATCTGACCTTGTTAACTATAATCCGATTGCGGAAAAGCACGTCAATGGGACGATGTCA  2712

Query  121   CTGGCTGAGCTTAGCGCGGCCGCGCTACAGTACAGCGATAACGTGGCGATGAATAAGCTG  180
             ||||||||||||||||||||||||||||||||||||||||||||||||||||||||||||
Sbjct  2713  CTGGCTGAGCTTAGCGCGGCCGCGCTACAGTACAGCGATAACGTGGCGATGAATAAGCTG  2772

Query  181   ATTGCTCACGTTGGCGGCCCGGCTAGCGTCACCGCGTTCGCCCGACAGCTGGGAGACGAA  240
             ||||||||||||||||||||||||||||||||||||||||||||||||||||||||||||
Sbjct  2773  ATTGCTCACGTTGGCGGCCCGGCTAGCGTCACCGCGTTCGCCCGACAGCTGGGAGACGAA  2832

Query  241   ACGTTCCGTCTCGACCGTACCGAGCCGACGTTAAACACCGCCATTCCGGGCGATCCGCGT  300
             ||||||||||||||||||||||||||||||||||||||||||||||||||||||||||||
Sbjct  2833  ACGTTCCGTCTCGACCGTACCGAGCCGACGTTAAACACCGCCATTCCGGGCGATCCGCGT  2892

Query  301   GATACCACTTCACCTCGGGCAATGGCGCAAACTCTGCGGAATCTGACGCTGGGTAAAGCA  360
             ||||||||||||||||||||||||||||||||||||||||||||||||||||||||||||
Sbjct  2893  GATACCACTTCACCTCGGGCAATGGCGCAAACTCTGCGGAATCTGACGCTGGGTAAAGCA  2952

Query  361   TTGGGCGACAGCCAACGGGCGCAGCTGGTGACATGGATGAAAGGCAATACCACCGGTGCA  420
             ||||||||||||||||||||||||||||||||||||||||||||||||||||||||||||
Sbjct  2953  TTGGGCGACAGCCAACGGGCGCAGCTGGTGACATGGATGAAAGGCAATACCACCGGTGCA  3012

Query  421   GCGAGCATTCAGGCTGGACTGCCTGCTTCCTGGGTTGTGGGGGATAAAACCGGCAGCGGT  480
             ||||||||||||||||||||||||||||||||||||||||||||||||||||||||||||
Sbjct  3013  GCGAGCATTCAGGCTGGACTGCCTGCTTCCTGGGTTGTGGGGGATAAAACCGGCAGCGGT  3072

Query   481  GGCTATGGCACCACCAAC  498
             ||||||||||||||||||
Sbjct  3073  GGCTATGGCACCACCAAC  3090
```

## D. BlastX best hit of *ctx-M-15.2*

```
Query  1    AVLLRSESEPNLLNQRVEIKKSDLVNYNPIAEKHVNGTMSLAELSAAALQYSDNVAMNKL  180
            AVL +SESEPNLLNQRVEIKKSDLVNYNPIAEKHVNGTMSLAELSAAALQYSDNVAMNKL
Sbjct  12   AVLKKSESEPNLLNQRVEIKKSDLVNYNPIAEKHVNGTMSLAELSAAALQYSDNVAMNKL  71

Query  181  IAHVGGPASVTAFARQLGDETFRLDRTEPTLNTAIPGDPRDTTSPRAMAQTLRNLTLGKA  360
            IAHVGGPASVTAFARQLGDETFRLDRTEPTLNTAIPGDPRDTTSPRAMAQTLRNLTLGKA
Sbjct  72   IAHVGGPASVTAFARQLGDETFRLDRTEPTLNTAIPGDPRDTTSPRAMAQTLRNLTLGKA  131

Query  361  LGDSQRAQLVTWMKGNTTGAASIQAGLPASWVVGDKTGSGGYGTTN  498
            LGDSQRAQLVTWMKGNTTGAASIQAGLPASWVVGDKTGSGGYGTTN
Sbjct  132  LGDSQRAQLVTWMKGNTTGAASIQAGLPASWVVGDKTGSGGYGTTN  177
```

**Figure S2:** BlastN and BlastX alignment of the newly identified *ctx* alleles to their best matching genes (variant nucleotides or amino acids are colored in red)

## REFERENCES:

1 Liebana, E. *et al.* Characterization of beta-lactamases responsible for resistance to extended-spectrum cephalosporins in Escherichia coli and Salmonella enterica strains from food-producing animals in the United Kingdom. *Microb Drug Resist* **10**, 1-9, doi:10.1089/107662904323047745 (2004).

2 Batchelor, M. *et al.* bla(CTX-M) genes in clinical *Salmonella* isolates recovered from humans in England and Wales from 1992 to 2003. *Antimicrob. Agents Chemother.* **49**, 1319-1322, doi:10.1128/aac.49.4.1319-1322.2005 (2005).

3 Woodford, N., Fagan, E. J. & Ellington, M. J. Multiplex PCR for rapid detection of genes encoding CTX-M extended-spectrum (beta)-lactamases. *J. Antimicrob. Chemother.* **57**, 154-155, doi:10.1093/jac/dki412 (2006).

4 Zong, Z., Partridge, S. R., Thomas, L. & Iredell, J. R. Dominance of blaCTX-M within an Australian extended-spectrum beta-lactamase gene pool. *Antimicrob. Agents. Chemother.* **52**, 4198-4202, doi:10.1128/aac.00107-08 (2008).

5 Saladin, M. *et al.* Diversity of CTX-M beta-lactamases and their promoter regions from Enterobacteriaceae isolated in three Parisian hospitals. *FEMS Microbiol. Lett.* **209**, 161-168 (2002).
